# Haptic Perception of Object Curvature in Parkinson's Disease

**DOI:** 10.1371/journal.pone.0002625

**Published:** 2008-07-09

**Authors:** Jürgen Konczak, Kuan-yi Li, Paul J. Tuite, Howard Poizner

**Affiliations:** 1 Human Sensorimotor Control Laboratory, University of Minnesota, Minneapolis, Minnesota, United States of America; 2 Department of Neurology, University of Minnesota, Minneapolis, Minnesota, United States of America; 3 Institute for Neural Computation, University of California San Diego, San Diego, California, United States of America; The Rockefeller University, United States of America

## Abstract

**Background:**

The haptic perception of the curvature of an object is essential for adequate object manipulation and critical for our guidance of actions. This study investigated how the ability to perceive the curvature of an object is altered by Parkinson's disease (PD).

**Methodology/Principal Findings:**

Eight healthy subjects and 11 patients with mild to moderate PD had to judge, without vision, the curvature of a virtual “box” created by a robotic manipulandum. Their hands were either moved passively along a defined curved path or they actively explored the curved curvature of a virtual wall. The curvature was either concave or convex (bulging to the left or right) and was judged in two locations of the hand workspace–a left workspace location, where the curved hand path was associated with curved shoulder and elbow joint paths, and a right workspace location in which these joint paths were nearly linear. After exploring the curvature of the virtual object, subjects had to judge whether the curvature was concave or convex. Based on these data, thresholds for curvature sensitivity were established. The main findings of the study are: First, 9 out 11 PD patients (82%) showed elevated thresholds for detecting convex curvatures in at least one test condition. The respective median threshold for the PD group was increased by 343% when compared to the control group. Second, when distal hand paths became less associated with proximal joint paths (right workspace), haptic acuity was reduced substantially in both groups. Third, sensitivity to hand trajectory curvature was not improved during active exploration in either group.

**Conclusion/Significance:**

Our data demonstrate that PD is associated with a decreased acuity of the haptic sense, which may occur already at an early stage of the disease.

## Introduction

The major symptoms of Parkinson's disease (PD) affect the motor system and lead to problems in movement initiation and movement speed. Yet, a growing body of research demonstrates that PD is also associated with an array of sensory or perceptual deficits, such as impairments in olfactory function, tactile discrimination and weight perception [1]–[5]. Evidence that kinaesthesia is especially affected in PD comes from experiments showing that PD patients perform poorly in tasks requiring matching, estimation or memorization of joint positions [6]–[8]. In addition, psychophysical studies have demonstrated that PD patients experience deficits in limb position and passive motion sense even in the early stages of the disease [9]–[13]. The fact that limb position sense is also impaired in other movement disorders such as focal dystonia [14] underlines the notion that kinaesthetic deficits are not specific to PD, but that other diseases affecting the cerebro-basal ganglia system have a detrimental effect on kinaesthesia as well.

Little is known about how haptic perception is affected by PD. The term haptic perception refers to an individual's sensibility to its adjacent surroundings by the use of his body [15]. This notion is closely linked to “active touch” which relies on the integration of proprioceptive, tactile, and pressure cues in conjunction with information gathered from efferent motor commands (motor plans). Previous studies suggested that the integration between visual information, proprioceptive information and motor commands may become impaired by PD [16]. However, there is a paucity of psychophysical studies which systematically examined the ability of PD patients to integrate somatosensory cues about limb motion and forces which form the basis of haptic perception.

This study evaluated the acuity of haptic perception in PD patients who were in the early to middle stages of disease (mild to moderate severity). Moving one's hand along a curved path provides a method for investigating the acuity of the haptic sense. Recent studies examining haptic perception have used a two-joint robot manipulandum to create curved “virtual walls” in free space which healthy subjects explored by moving a handle attached to the end of the robotic arm [17], [18]. We used a similar but slightly modified procedure in which we reduced the availability of tactile and pressure cues, thereby requiring participants to rely almost exclusively on limb proprioception in order to make judgments about their hand path curvature. In order to differentiate the effect of active exploration versus the sensation of passive limb motion, participants were tested under two conditions. In one condition they actively explored the curvature of a virtual wall; in the second condition the robot moved a subject's hand passively along curved paths.

Specifically, this study addressed the following questions: a) Do PD patients have decreased sensitivity to detect the curvature of their own hand paths when they cannot rely on vision? b) Is curvature sensitivity differentially affected in PD during active versus passive limb motion? Finding that curvature sensitivity is decreased in PD would indicate an impaired mechanism of processing and integrating proprioceptive and tactile information. In addition, demonstrating that curvature sensitivity is less affected in PD during active than passive motion would indicate that kinematic information derived from an efference copy of the underlying motor commands may be used to compensate for a loss in proprioceptive or tactile sensitivity.

## Methods

### Subjects

Eleven patients with PD participated in the study (ages: 48–70 years; mean±SD ages: 60.18±7.72 years; four females and seven males; all right handed with initial right side-onset of disease). Eight age-matched healthy subjects between 50–76 years without neurological disease served as a control group (mean±SD ages: 62.50±7.72 years; one female and seven males; all right handed). All participants were dominant right-handers based on the results of the Edinburgh Handedness Inventory [19]. Informed written consent was obtained from participants prior to testing. The study was approved by the Institutional Review Board of the University of Minnesota.

PD patients were recruited from the movement disorders outpatient clinic at the University of Minnesota. The eleven patients were clinically diagnosed as having idiopathic PD [20]. Nine patients were evaluated while taking and obtaining an optimal response from their routine antiparkinsonian medications (ON state). The other two subjects had never taken any antiparkinsonian medications up to the time of testing. Prior to testing each patient underwent a clinical examination to determine the severity of disease using the Unified Parkinson's Disease Rating Scale (UPDRS), which revealed they were in mild or moderate stages of the disease: UPDRS mean total score±SD: 42.9±13.6. In order to assess general cognitive function, the Mini-Mental State Examination (MMSE) [21] was administered to PD and healthy control subjects. The PD patients fell within the normal range. Their MMSE mean score was 29.6 out of a possible score of 30. Additionally, a neurological examination did not show signs of peripheral nerve dysfunction. Daily doses of medication were standardized by computing the levodopa-equivalent dosage. Each patient's levodopa equivalent dose and other relevant patient characteristics are summarized in Table 1. The right arm was more affected than the left in each PD subject, and the right arm was tested in the PD group. None of the patients exhibited a substantial hand or arm tremor during testing.

**Table 1:** Characteristics of Parkinson's disease patients.

N	Age	Gender	Handedness	Disease duration (years)	UPDRS				Levodopa equivalent dose (mg/diem)	Medication
					Total (max = 199)	Mentation, behavior,mood (max = 16)	ADL (max = 52)	Motor (max = 108)		
1	70	F	17	4	55	2	10	40	300	L
2	67	F	16	0.58	45	4	9	31	300	L
3	66	M	15	3	48	4	10	33	12.5	R
4	51	M	18	5	50	4	9	33	420	L, P
6	63	M	14	12	51	2	11	36	706	L, P
7	54	F	17	6	21	1	1	12	840	L, R
8	70	M	20	6	26	1	4	17	1150	L, R
9	54	M	20	3	39	1	5	33	0	none
10	48	F	18	8	63	3	17	43	300	P
11	58	M	20	7	34	1	4	24	1285	L, R
12	61	M	17	1	57	0	13	43	0	none

Gender: M = male; F = female; Handedness: according to the Edinburgh Handedness Inventory (scores range from 20 to −20, 20 = right handed, −20 = left handed) [19]; UPDRS: United Parkinson's Disease rating scale; ADL: Activities of daily living; L: Levodopa; P: Pramipexole; R: Ropinirole. Levodopa equivalent dose: 100 mg standard levodopa = 125 mg sustained-release levodopa or 1.5 mg pramipexole or 6 mg ropinirole or 10 mg bromocriptine or 1 mg pergolide; Medication: L = Levodopa; P = Pramipexole; R = Ropinirole.

### Apparatus and Procedure

**Figure 1:**
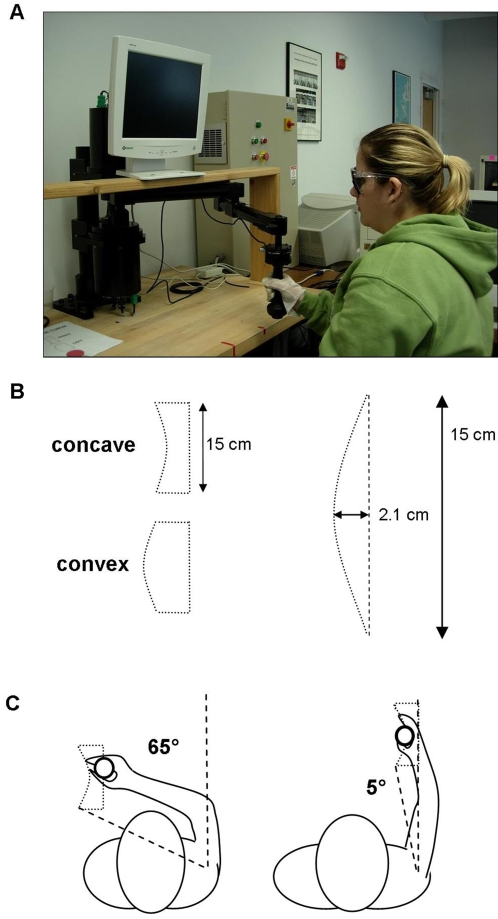
A. Experimental setup. Subjects moved the handle of the MIT Manus robot manipulandum. Vision was occluded and haptic information from gripping the handle was reduced by wearing a glove made of low friction material. B. Movements were made within the boundaries of a 15×5 cm box, where its left side was curved either concave or convex. The manipulandum generated necessary boundary forces. During the passive condition the hand was moved along the boundaries of the box. Maximum curvature translated to a 2.1 cm deviation from a straight path. C. This graph depicts the approximate positions of the virtual boxes in the hand workspace. Transverse shoulder angle at the lower left corner was approximately 65° for the box in the subject's left hemi-workspace and 5° in the right hemi-workspace.

Participants moved the handle of a two-joint robotic manipulandum (*Interactive Motion Technologies, InMotion2*). They sat facing the robot holding the robot handle above waist level. Vision was blocked by having subjects wear opaque glasses. To reduce haptic information, participants wore a synthetic gauze glove, which reduced friction between skin and handle, and thus minimized the amount of tactile and pressure-related haptic information (see Fig. 1A). Consequently, participants had to rely primarily on joint proprioception to detect the curvature of their hand trajectories. This reliance on proprioception was confirmed in our pilot studies by subjects indicating that they concentrated on the elbow/shoulder joint configuration to detect the curvature of the hand path. In the *active* movement condition, the robot was programmed to generate boundary forces (stiffness: 2500 N/m, damping: 500 N/m/s) that kept the participant's hand within a virtual box (5 cm×15 cm) with a curved left wall. At the boundary, the experienced resistance was comparable to hitting a wall. Subjects actively moved their hand within the box. In a second condition, the subject's hand was moved *passively* at a constant velocity of 0.033 m/s by the robot along a path with the same dimensions as the box in the active condition. Curvature of the left side of the *active* or *passive* box was either concave or convex with curvature values ranging from 7 to −7 m^−1^. A curvature of 7 m^−1^ translated to a 2.1 cm deviation from the straight path (see Fig. 1B). In the active condition, subjects were instructed to move in the clockwise direction along the virtual walls until they were ready to make a judgment (typically 1–5 rotations). Subjects were instructed to move at approximately the same speed as during the passive movement condition. We did not use auditory cues to enforce appropriate speed, because it distracted participants from focusing on the virtual curvature. At the end of each trial subjects had to indicate whether the hand trajectory traversing the curved virtual wall of the box was curved “to the right” (concave) or “to the left” (convex). That is, we applied standard forced-choice paradigm not allowing for judgments such as “I don't know” or I cannot tell”.

**Figure 2:**
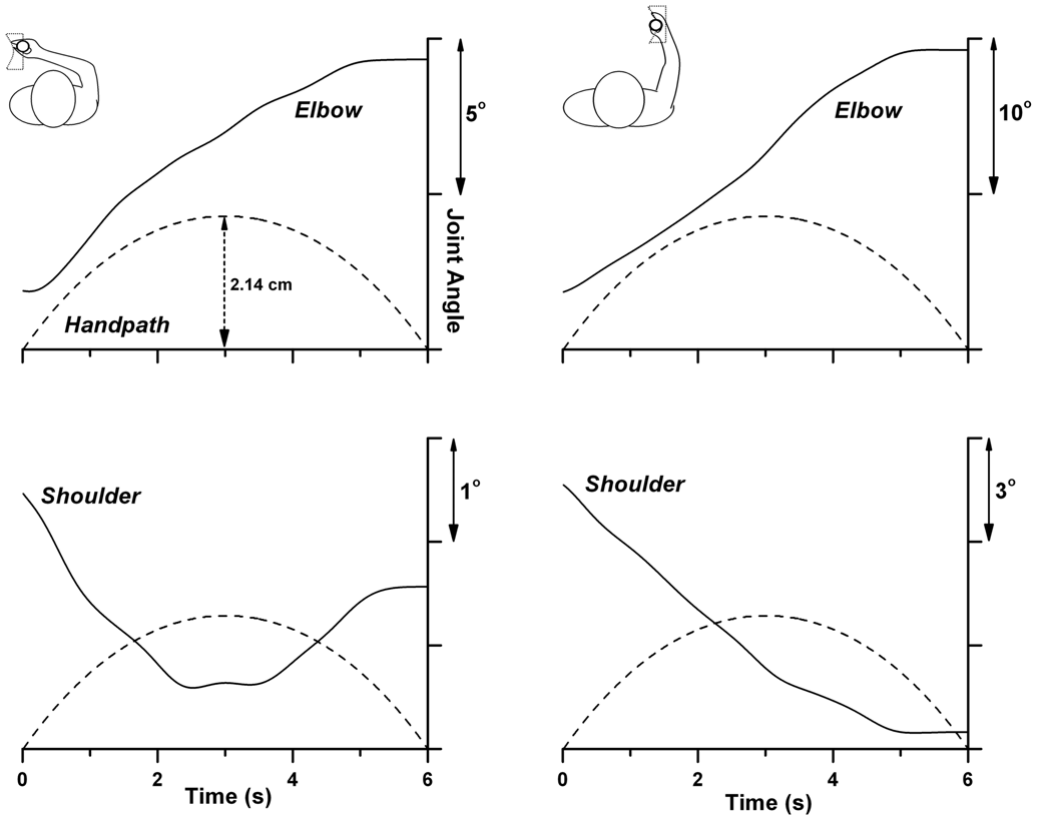
Proximal joint paths and hand path during exploration of a convex curved virtual contour in the two hemi-workspaces. Shown are exemplar shoulder and elbow joint angles and the hand path of one subject while her hand was moved passively by the robot along a convex shaped contour with the curvature of 7.05 m^−1^ (max. leftward displacement: 2.1 cm). Note that hand motion following the virtual contour in the right hemi-workspace required concurrent shoulder and elbow extension (i.e., a nearly linear change in joint angle), while contour exploration in the left hemi-space required a movement reversal at the shoulder (i.e., flexion followed by extension), which meant that hand path and shoulder angular path were highly correlated (r = −0.94).

Movements were made in two boxes that were located in the left or right half of the individual's hand workspace (see Fig. 1C). One box was located 13 cm to the right of the subject's midline, the second box 13 cm to left of it. The center of left virtual box was located closer to a participant's trunk, while the center of the right box was placed more distally. The distance between the centers of both boxes was 22 cm along the sagittal axis. The presented curvatures of the virtual box did not vary between the two workspace locations. However, the joint angular paths and the joint amplitudes differed between the two locations. For the right virtual box hand motion along the curved side of the box was associated with concurrent shoulder and elbow flexion, i.e., a linear increase in shoulder and elbow angles, while in the left box the curved hand path was associated with “curved” angular joint trajectories, meaning that the proximal joint movements were associated with movement reversals, i.e. shoulder flexion and extension (see Fig. 2). The Pearson product-moment correlations between hand path and shoulder angle were computed as r = −0.94 when moving in the left workspace, and r = 0.21 for motion in the right hemi-workspace. The correlations between hand path and elbow angle were computed as r = −0.07 when moving in the left workspace, and r = 0.21 for motion in the right hemi-workspace along the convex curvature.

At the beginning of testing the initial curvature was set to either 7 to −7 m^−1^. After each trial the participant indicated verbally whether the left wall of the box was concave or convex (“curved right” or “curved left”). Based on this judgment, the curvature of the virtual wall was adjusted in the subsequent trial using an adaptive staircase procedure. We used an adaptive staircase algorithm proposed by Kesten called *accelerated stochastic approximation* ([22]; see [23] for a review). Using this method the stimulus was not increased or decreased by a fixed amount, but depended on the subject's response. The initial step size was set to 0.75 m^−1^. The curvature value of the presented curvature changed only when a shift in the response category occurred (from correct to incorrect or vice versa). The implementation of the algorithm led to asymmetric step sizes. That is, as the subject approached his/her perceptual threshold the presented step size of the stimulus became smaller for a correct answer and increased for an incorrect answer. For example, when a subject correctly identified a convex curvature at 4 m^−1^, the subsequent curvature value decreased by 0.75 m^−1^ in the following trial. However, if the subject's response was incorrect the curvature was increased to 4.75 m^−1^ and then decreased by 2/3 of last step size in the subsequent trial, effectively approaching the incorrect response value in smaller increments. The method guaranteed that the sequence of curvature values converged to the threshold almost monotonically for all conditions (left vs. right work space; passive vs. active motion). This allowed for a more precise determination of the threshold when compared to a fixed staircase paradigm [23]. A total of 200 trials were administered, with 100 trials for each of the active and passive movement conditions. Within each movement condition 50 trials were performed in a blocked presentation in the left and 50 trials in the right hemi-workspace.

### Measurements

For each trial, we recorded the location of the virtual box, the curvature value of its left wall, and the associated judgment of the subject. Data analysis was performed using customized algorithms based on the MATLAB technical programming language. We computed the curvature *sensitivity threshold* as follows:
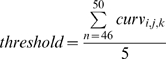
(1)where *curv* is the presented curvature stimulus determined by three levels (i: 1 = convex, 2 = concave; j: 1 = left workspace, 2 = right workspace; k: 1 = active motion, 2 = passive motion) for the 46^th^ to 50^th^ trial of each block. That is, we obtained a threshold for detecting convex and one for detecting concave curvatures.

**Figure 3:**
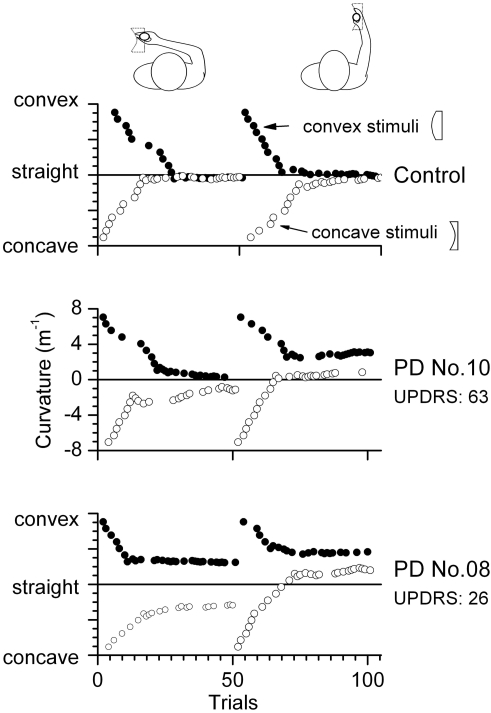
Exemplar performance of 3 participants during the passive movement condition. Each data point indicates a presented curvature value. Two blocks of trials were administered - one in the right hemi-workspace, and one in the left hemi-workspace. Initial curvature values were ±7.05 m^−1^. The two staircase procedures were intertwined. Thus, depending on the subject's answer, the curvature could switch between convex and concave within a given block. The black circles (•) represent the data for the staircase judging convex curvature (“curved to the left”); the open circles (○) indicate the concave staircase (“curved to the right”). In the control subject, the presented convex and concave curvatures converged around zero at the end of each block. In PD patient No. 10 curvature values did not fully converge at the end of each block, especially for motion in the left hemi-workspace. In PD patient No. 8 sensitivity to curvature was markedly lowered. The correctly identified hand trajectory curvatures for this patient leveled off at approximately ±3 m^−1^.

The computation of a single sensitivity threshold, which is customary for determining a single detection threshold, was not a sensible procedure for many PD data sets. Using the data of both staircase procedures to compute a single threshold would require both staircase procedures to converge to a common value. However, the data of many PD patients did not show the necessary convergence (see Figure 3). For each subject, eight sensitivity thresholds were computed based on the 2×2×2 experimental design (left or right hemi-workspace, active or passive movement and concave or convex curvature type).

### Statistical analysis

All reported statistics were computed using SAS 9.1 software. Because we had no data about the true variance and distribution of each population, we performed non-parametric Wilcoxon signed-rank tests to eliminate the assumptions for population distributions required for parametric tests.

## Results

### Association of proximal joint amplitudes with curved hand paths

Absolute joint amplitudes naturally depended on the arm anthropometrics. To obtain information about the range of arm joint amplitudes while the hand moved along the curved virtual wall, we collected electrogoniometer measurements during trials from a tall (184 cm) and a small (162 cm) participant. We found that the ranges of their shoulder joint angles in the right hemi-workspace were 3.8 to 7.8 times larger when compared to the left workspace (angular range for small subject: 7.3° vs. 1.9°, tall subject: 4.5° vs. 0.6°). Similarly, elbow joint amplitudes were approximately twice as large in the right hemi-workspace in contrast to the left hemi-workspace (angular range for small subject: 15.7° vs. 7.7°, tall subject: 12.8° vs. 6.0°). Since the hand path did not differ between the two workspaces, the larger joint amplitudes implied that the joint motions had to be faster. When compared to the left hemi-workspace mean shoulder angular velocity increased seven- to tenfold, while elbow angular velocity approximately doubled.

### Judging virtual curved object curvature

For each movement type (active vs. passive) and in each hemi-workspace, subjects were presented with two series of stimuli, one starting with left curved curvatures (convex; descending staircase) and one with right curved curvatures (concave; ascending staircase). To appreciate the observed range of performance between subjects, Figure 3 shows typical data series of three participants, one healthy control subject and two patients. This figure illustrates that for the control subject, sensitivity for convex as well concave curvature series converged close to 0 m^−1^. However, only four of the 11 PD patients (36%) revealed such convergence of curvature values in at least one of the four test conditions.

**Figure 4:**
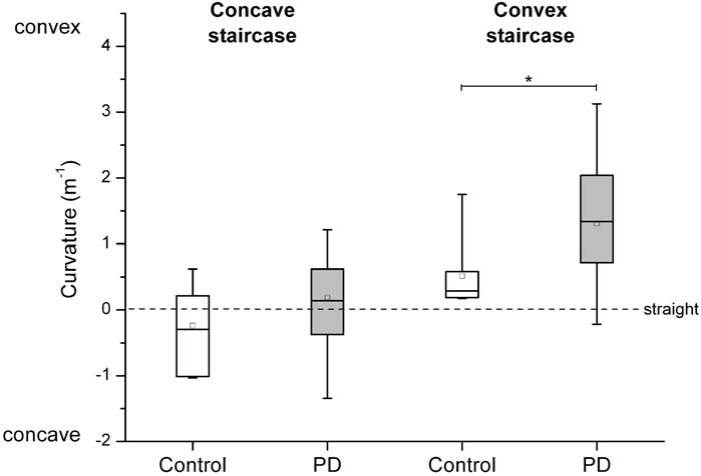
Distribution of curvature sensitivity of each group for the concave and convex staircase series. The box indicates the 1^st^ and 3^rd^ quartile, the line across the box represents the median, and the square inside the box is the mean. Whiskers represent the 1% and 99% percentile. The dashed line indicates a straight contour (curvature = 0 m^−1^). The asterisk (*) indicates a significant group difference (p<0.001).

Based on the two staircase series, we computed the thresholds for each staircase stimulus series for each subject. The respective group data for the convex and concave staircases are presented in Figure 4. Within-group comparisons revealed that curvature thresholds for the convex and concave staircase series were significantly different from each other in each group (controls: z = −2.20, p = 0.0141; PD: z = −3.75, p<0.0001), meaning that both groups had a higher haptic acuity for concave curvatures.

### Sensitivity to concave curvature was normal in the PD patient group

The median sensitivity thresholds for detecting concave curvatures (“curved to the right”) were 0.16 m^−1^ in the control group and 0.14 m^−1^ in the PD group (see Figure 4). A Wilcoxon signed-rank test on detection thresholds for concave curvatures did not yield a significant difference for group (p>0.05). In addition, no significant effects for movement type and hemi-workspace were found. It is noteworthy that both the PD and the control groups had lower sensitivity thresholds for judging concave curvatures when compared to convex curvatures (see below).

### Sensitivity to convex curvature was lower in the PD patient group

**Figure 5:**
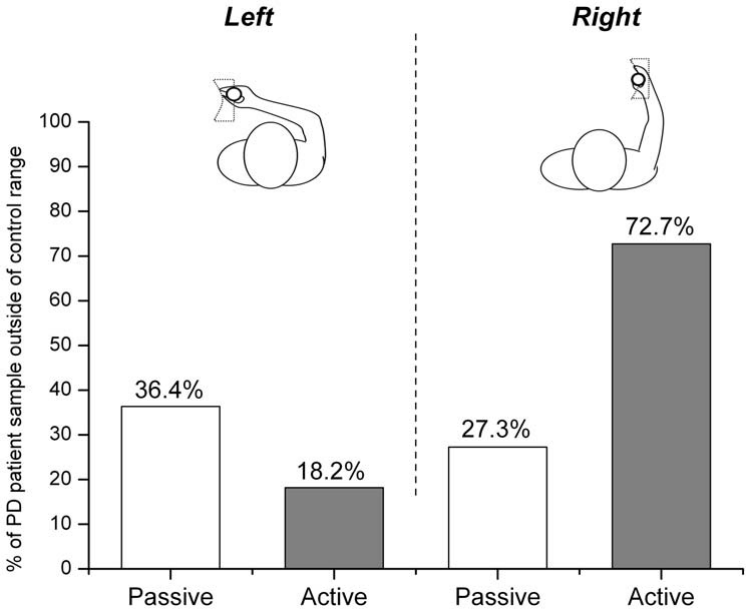
Percentage of PD patients, whose detection thresholds for convex curvature contours were outside the range of the control group in the respective experimental conditions.

The median thresholds for detecting convex curvatures were 0.30 m^−1^ in the control group and 1.33 m^−1^ in the PD group. A Wilcoxon signed-rank test on sensitivity thresholds for convex curvatures yielded a significant effect for group, z = −3.10, p = 0.001 (see Figure 4). Within the PD group, 9 out of 11 patients (82%) revealed sensitivity thresholds outside the range of the control group in at least one of the four test conditions. Five out of 11 patients (45%) exhibited curvature thresholds outside the normal range in at least 2 conditions. Figure 5 presents the percentage of PD patients whose sensitivity thresholds fell outside the range of the thresholds seen in the control group participants in each of the four conditions.

### Convex curvature detection thresholds were larger in the right hemi-workspace

**Figure 6:**
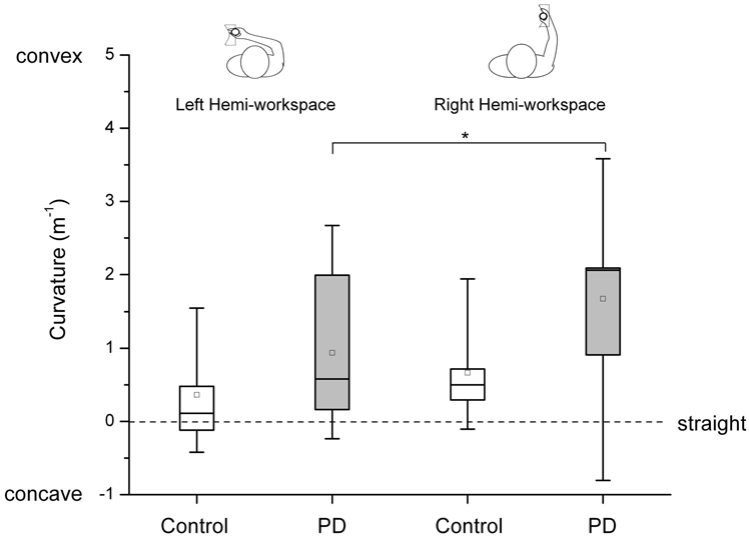
Distribution of curvature sensitivity of each group for the right and left hemi-workspace. The box indicates the 1^st^ and 3^rd^ quartile, the line across the box represents the median, and the square inside the box is the mean. Whiskers represent the 1% and 99% percentile. The dashed line indicates a straight contour (curvature = 0 m^−1^). The asterisk (*) indicates a significant group difference (p<0.05).

Moving within the right box required larger amplitudes of shoulder and elbow motion and was associated with more linear joint paths. In comparison, moving within the left box gave rise to more complex sinusoidal joint paths. This difference in joint path patterns affected curvature sensitivity in the control as well as the patient group. When compared to the right hemi-workspace, the median sensitivity in the left hemi-workspace was at least twice as high for both groups (controls: 55.69%, PD: 28.17%). The corresponding Wilcoxon signed-rank test yielded a significant effect for hemi-workspace for convex curvature thresholds in PD group (z = 1.70, p = 0.04). However, no significant difference was found between right and left hemi-workspace for the control group (see Fig. 6).

### Effect of active movement on convex curvature detection

Comparing the curvature sensitivity during active movement with the sensitivity during passive motion showed that the median thresholds for detecting curvature were generally lower in the passive condition in both groups. The median thresholds for detecting convex curvatures were 0.16 m^−1^ during passive movement and 0.49 m^−1^ during active movement in the control group. In the PD group, the median thresholds for detecting convex curvatures were 0.71 m^−1^ during passive movement and 1.32 m^−1^ during active movement. Although the median thresholds were larger during active movement, the corresponding Wilcoxon signed-rank test did not yield a significant effect for movement type for convex curvature thresholds (p>0.05).

### Correlation between convex curvature, medication and clinical scores

To examine the relationship between curvature detection and medication we performed a set of correlation analyses. The Pearson product-moment correlation between convex curvature threshold and levodopa equivalent dosage yielded a value of r = 0.305 (p = 0.044). The correlation was significant, but meant that levodopa dosage explained less than 10% of the variance in curvature threshold. In a second step we investigated how clinical markers of disease severity were associated with thresholds of curvature detection. The correlations between curvature thresholds and UPDRS_total_ and UPDRS_motor_ scores were computed to be r = −0.22 and r = −0.19, respectively. Neither correlation was statistically significant.

## Discussion

This study examined whether the sensitivity to perceive the curvature of one's hand trajectory is affected by PD. The haptic perception of geometric properties such as the curvature of a curvature is based on the availability of somatosensory cues about the motions and forces experienced during exploratory actions. Signals from proprioceptive receptors and from cutaneous and mechanoreceptors provide the primary sources of information for this perception. Recent research suggests that the perception of actual hand trajectories is likely not derived from sensing force feedback, but is inferred from proprioceptive feedback [24], which implies that proprioceptive information plays a primary role in judging hand path trajectories. When the limb is moved passively, the reliance on kinaesthetic information from shoulder and elbow joints for judging hand path curvature is likely increased, especially when vision is absent and tactile information from the palmar surface of the hand is reduced as it was the case in the current experiment.

The main findings of the study can be summarized as follows: First, PD reduces the sensitivity in perceiving hand path curvature. Second, healthy controls and the PD patients showed a decrement in curvature sensitivity when the accuracy of proprioceptive information was diminished due to an increased speed of joint rotations or because the curvature of the proximal arm joint paths was less correlated with the curved hand paths. This was the case when moving in the right hemi-workspace. Third, sensitivity to hand trajectory curvature was not improved during active movement in either the PD or the healthy control group.

### What aspects of kinaesthesia or haptic perception were examined?

In this experiment we asked subjects to judge the curvature of their own hand path. Humans use sensory information derived through vision, tactile sensation of the skin or kinaesthetic sensation of the position and movement of the joints to perceive the curvature of objects or the curvedness of one's movement trajectories. If vision is blocked and the availability of tactile information is reduced by wearing a glove made of a low friction material, as in our experiment, then humans need to rely mainly on proprioceptive information to make such judgments. Since the hand is the distal part of the arm, the perception of one's hand position and movement in space requires the processing and integration of proprioceptive information across the wrist, elbow and shoulder joints. Given that the afferents from mechanoreceptors in the hand were not fully blocked through local anesthesia in this study, tactile information from the skin could have contributed to the perception of hand path curvature. That is, the haptic perception of hand path curvature was likely based on two processes of sensory integration: The integration of proprioceptive information across several joints and the integration of multijoint proprioceptive information with information derived from tactile receptors of the fingers and palm.

With respect to kinaesthesia it is known that humans detect and can match joint angles with a maximum precision of around 1° [11], [25]–[27]. Recent studies documented that the acuity of the haptic sense compares well to vision [28]. When subjects were certain that their hand path was curved “out” or “in” their shoulder angles differed by just 0.2° at the mid arc [29]. Another way of illustrating the sensitivity of humans to detect even small deviations from a straight hand path, consider that in this study a curvature detection threshold of 0.5 m^−1^ implies that a lateral deviation of 1.4 mm was detected over a movement amplitude of approximately 15 cm.

With respect to the type of curvedness, we found that subjects were in general more sensitive in judging concave curvatures (“curved to the right”). This perceptual bias for convex curved curvature has been reported in earlier studies [18], [29], and we found this bias expressed in the control group as well as the PD patient group. Since the available tactile information from holding the robot handle was altered between the different curvature types, a possible explanation for this observed bias for convex curvatures may be that proximal joint paths yielded more curvature relevant information under this condition. However, to fully support such claim recordings of the three-dimensional kinematics of the arm joints are needed, which we were unable to do in this study.

### Curvature sensitivity is reduced in PD

A main finding of this study is that the thresholds for detecting convex hand path curvature (“curved to the left”) were elevated in PD patients. Such loss in sensitivity is likely common in PD, considering that 82% of our PD patients revealed detection thresholds outside the control group range in at least one experimental condition and 5 out of 11 patients showing reduced haptic acuity in two or more of the four test conditions. The median detection threshold for convex curvature was increased by 343% when compared to healthy controls (see Fig. 4). Knowing that the patients in this study had mild to moderate disease severity, our finding implies that this perceptual impairment may occur at the early stages of the disease.

We then investigated whether the differences in perceptual performance were related to disease duration, disease severity or to medication. Using the clinical UPDRS scores as markers of disease severity we found no strong association between disease severity, disease duration and detection thresholds. This stands in contrast to previous research reporting that disease severity as measured by UPDRS correlated strongly with a loss of sensitivity in the sole of the foot [30] or in detecting changes in limb position [11]. Several reasons may account for the failure to document a close relationship between curvature sensitivity and disease severity or disease duration: First, the determination of the exact disease onset remains difficult in PD. Our estimates were based on patient reports or first clinical diagnosis, which can only be regarded as approximations of the true disease onset. Second, the clinical UPDRS scores may have provided too coarse of a measure for disease severity. Third, the size of the patient sample may have been too small to yield significant correlations, although other studies with similar sample sizes have reported highly significant correlations between proprioceptive thresholds and disease severity in PD [11].

With respect to the role of medication, we found a small, but significant positive correlation between levodopa equivalent dosage and convex curvature thresholds (r = 0.3). This hints, as other studies have suggested [8], that levodopa may play some role in enhancing the kinaesthetic deficits in PD. However, our study was not designed to examine the effect of levodopa on haptic perception, because the patients were not studied in their “on” and “off” states. It is noteworthy that two patients who had never taken levodopa up to that time of testing showed highly elevated detection thresholds, which implies that the disease and not levodopa was responsible for the decrease in haptic acuity in these patients

### Reduction of curvature-relevant proprioceptive information reduced curvature sensitivity in PD patients and controls

We presented the virtual curved walls in two different locations of a person's workspace. Given the geometry of the arm and the task constraints moving within the two different hemi-workspaces gave rise to different joint paths and joint velocities. Moving within the right box was mainly associated with simple extension at both the shoulder and elbow and higher joint angular velocities. In comparison, moving within the left box yielded more sinusoidal joint paths that corresponded more closely to the curvature of the hand path. That is, proprioceptive information derived from the proximal joints contained less information about the curvedness of the virtual wall when participants moved within the right hemi-workspace. In addition, joints rotated faster in the right when compared to the left location, even though hand motion had a similar speed. Given the higher speed and the more linear joint paths of the right workspace movements the processing of proprioceptive information was likely less accurate and we expected that subjects would be less sensitive in that condition.

This expectation was confirmed when we found that for motion in the right box, the curvature sensitivity was approximately reduced by a factor of 2 for the control as well as for the patient group (see Fig. 6). This means that the effect of the “impoverished” proximal joint proprioceptive information on haptic perception of hand curvature was similar in PD patients and healthy individuals. In other words, the relative change in curvature sensitivity, when switching from the left to the right workspace, was comparable for both groups. The result is noteworthy because the curvature sensitivity in absolute terms was reduced in most PD patients

### Active exploration cannot overcome the loss in passive motion sensitivity in PD

We provided PD patients with the opportunity for active exploration of a virtual arc to investigate whether the active generation of movement would help restore a potential loss in passive motion sensitivity. The rationale for measuring curvature sensitivity during active movement was that PD patients could use information derived from an efference copy of their motor commands to predict the curvedness of their hand trajectories via the use of an internal forward dynamics model [31]. Presumably, processes in the cerebro-cerebellar loop facilitate the prediction of sensory consequences of intended movement [32], which are believed to be intact in PD. In addition, it is known that healthy individuals exhibit lower variable endpoint errors during active reaching motions when compared to passive motion, which indicates that kinaesthetic acuity may be higher during self-generated, goal-directed action [33]. We could not confirm that active movement helped to improve kinaesthetic or haptic sensitivity in PD.

### Concluding remarks

The results from this experiment add to the growing body of literature indicating that PD is associated with a loss of proprioceptive function. Previous studies documented that limb position and passive motion sense are affected in PD at the level of a single joint [11], [13]. Here we document that the proprioceptive impairment extends to multijoint motion and impacts the haptic perception of object curvatures. The functional consequences of a reduced acuity in the kinaesthetic perception of distal limb motion are not trivial, but very likely contribute the observed motor symptoms in PD. For example, a motor control system that has only noisy data about limb motion and limb position available will have difficulty in planning accurate, fast movements. It will have problems placing distal limb segments like feet and hands in the task-appropriate position at the right time, which may lead to increased falling and to error prone fine motor control. On the background of a faulty proprioceptive system, the hypometric or dyskinetic movement trajectories that are so commonly observed in PD patients become understandable. This underlines the notion that PD is not a primary motor system disease, but with respect to motor behavior needs to be understood as a sensorimotor or perceptual-motor disease.
